# Knowledge of Antimicrobial Resistance among Veterinary Students and Their Personal Antibiotic Use Practices: A National Cross-Sectional Survey

**DOI:** 10.3390/antibiotics8040243

**Published:** 2019-11-29

**Authors:** Ismail A. Odetokun, Uduak Akpabio, Nma B. Alhaji, Khalid T. Biobaku, Nurudeen O. Oloso, Ibraheem Ghali-Mohammed, Asmau J. Biobaku, Victoria O. Adetunji, Folorunso O. Fasina

**Affiliations:** 1 Department of Veterinary Public Health and Preventive Medicine, University of Ilorin, Ilorin 240272, Kwara State, Nigeria; drghalimohd@gmail.com (I.G.-M.); abiobaku@yahoo.com (A.J.B.); 2 Department of Veterinary Public Health and Preventive Medicine, Michael Okpara University of Agriculture, Umudike 440109, Abia State, Nigeria; udiakpabio@yahoo.com; 3 Public Health and Epidemiology Unit, Niger State Veterinary Hospital, Bosso, Minna 920211, Niger State, Nigeria; nmabida62@gmail.com; 4 Department of Veterinary Pharmacology and Toxicology, University of Ilorin, Ilorin 240272, Kwara State, Nigeria; biobaku.kt@unilorin.edu.ng; 5 Epidemiology Section, Department of Production Animal Studies, Faculty of Veterinary Science, Onderstepoort Campus 0110, University of Pretoria, Pretoria 0110, South Africa; nurudeenoloso@gmail.com; 6 Department of Veterinary Public Health and Preventive Medicine, University of Ibadan, Ibadan 200284, Oyo State, Nigeria; vadetunji@gmail.com; 7 Department of Veterinary Tropical Diseases, Faculty of Veterinary Science, Onderstepoort Campus 0110, University of Pretoria, Pretoria 0110, South Africa; daydupe2003@yahoo.co.uk; 8 Emergency Centre for Transboundary Animal Diseases-Food and Agriculture Organization of the United Nations (ECTAD-FAO), Dar es Salaam 14111, Tanzania

**Keywords:** antimicrobial resistance, antibiotics use, veterinary students, knowledge, awareness, Nigeria

## Abstract

The challenge of antimicrobial resistance (AMR) is grave in developing countries. Antimicrobials are misused yet stakeholders’ contribution to antimicrobial stewardship is low. Veterinary students are future prescribers and their knowledge could influence progress in combating AMR; hence, there is a need to assess their knowledge, attitude, and awareness of AMR. A multi-institutional questionnaire was administered to undergraduates in Nigerian veterinary schools. It comprised demographics, own personal antibiotic usage, and knowledge, attitude, and awareness of AMR in humans and animals. Descriptive statistics and logistic regression were used for analyses. Of the 426 respondents, 39.2% reported personal antimicrobial use in the previous six months. Over 60% received knowledge scores lower than average and >87% requested more education on clinical use and prescriptions pre-graduation, monitored dispensing of antimicrobials, conducting AMR research, and confirmed link among human, animal, and environmental health. Less than 25% of respondents were aware of antimicrobial stewardship and global efforts/organizations for AMR. Final year students have 9-fold and 14-fold more satisfactory knowledge on antimicrobials in humans and animals compared with other students, respectively (*p* = 0.001). Final year students also have more knowledge (13×) and awareness of contributory factors (3×) on AMR (*p* = 0.001) than other students. Unsatisfactory knowledge on AMR issues exists among veterinary students yet willingness to improve was observed. Identified knowledge, attitude, and gaps in AMR awareness should be targeted by veterinary schools in Nigeria.

## 1. Introduction

Globally, antimicrobial resistance (AMR) is a complex public, animal, and environmental health problem, primarily fueled by inappropriate use of antimicrobials. The trends and problems associated with AMR are observed naturally in pathogenic and commensal or non-pathogenic bacterial organisms [1,2]. Currently, AMR is one of the greatest health threats that demands the most urgent attention in global health security. Antimicrobial abuse, misuse, and overuse in animals have devastating effects on humans, animals, and the environment, resulting in serious health and economic consequences [3,4]. In Africa, these consequences are often underreported due to inadequate surveillance data [5,6,7]. AMR is projected to be attributed with about 4.15 million human deaths per annum in Africa by the year 2050 if left to continue uncontrolled [6]. The understanding of barriers and enablers to effective control of AMR is poorly documented in Nigerian veterinary settings. Rational use of antimicrobials remains the main strategy for the prevention of AMR, and this can be achieved by changing the prescribers’ behavior and knowledge, right from the period of formal training. In most developing countries, particularly Nigeria, greater emphasis on AMR control has been placed on incidences in humans, with almost inexistent attention paid to animals (livestock, wildlife, and poultry industry as well as in veterinary clinical practices) until recently [3]. Discrepancies exist and the level of attention paid to the issue of AMR among the public, animal, and environmental health sectors differ. Hence, resource allocations available to combat AMR in Nigeria have wide discrepancies with consequences of threatened food security in addition to an increased AMR challenge. There is a need to understand the current knowledge and propose means to bridge the existing gap on the trend, status, and situation of AMR arising primarily from food animals and the environment [8,9].

Huge antimicrobial consumption and misuse are prevalent in some low- and middle-income countries, such as Nigeria [10,11]. Over 20 brands of commercially available antimicrobials are used as growth promoters in the Nigerian livestock industry alone [12], despite the issued ban by the National Agency for Food and Drug Administration and Control, the institution with the mandate to regulate antimicrobial inclusion in human foods. Antimicrobials are freely available in Nigerian markets, veterinary pharmacies, and agro-allied shops and could often be easily acquired over the counter without prescription by non-veterinarians, including pastoralist and nomadic herders [13]. Tetracyclines are the most commonly misused antibiotics [3,13,14]. Few studies have documented the irrational use of antimicrobials especially without prescription in the human medical practice in Nigeria [15,16]. This misuse was reportedly fueled by over-the-counter sale and access to unprescribed antimicrobials [17,18].

Specifically, veterinarians are very important stakeholders and play a vital role in the mitigation of AMR [8,19,20,21]. The education, awareness, and training of veterinary students is vital to the improvement of antimicrobial use patterns in farms, clinics, and hospitals as well as in many animal species [8]. Veterinary students are future prescribers and their knowledge could influence progress in combating AMR [8]. Inadequate exposure of veterinary students to the concept of AMR could affect their practice of drug administration and management after graduation [22,23,24]. Satisfactory knowledge levels of veterinary students on personal antibiotic administration (self-consumption), in addition to their perceptions, attitude, and knowledge of AMR could enhance their practice behaviors [8,25].

The scope of AMR in the academic training of undergraduate veterinary students in most Nigerian schools is not clearly defined. A previous survey of final year students in five conveniently selected veterinary schools in Nigeria indicated poor knowledge of AMR [26]. In this study, we conducted a cross-sectional survey on personal antimicrobial usage among students (year two to year six), studying for the degree of doctor of veterinary medicine (DVM) from 10 veterinary schools in the country. This is the first nationwide multi-institutional survey on the personal use of antimicrobials and knowledge, attitude, and awareness of AMR among veterinary undergraduate students in Nigeria.

## 2. Materials and Methods

### 2.1. Structure of the Target Population

**Figure 1:**
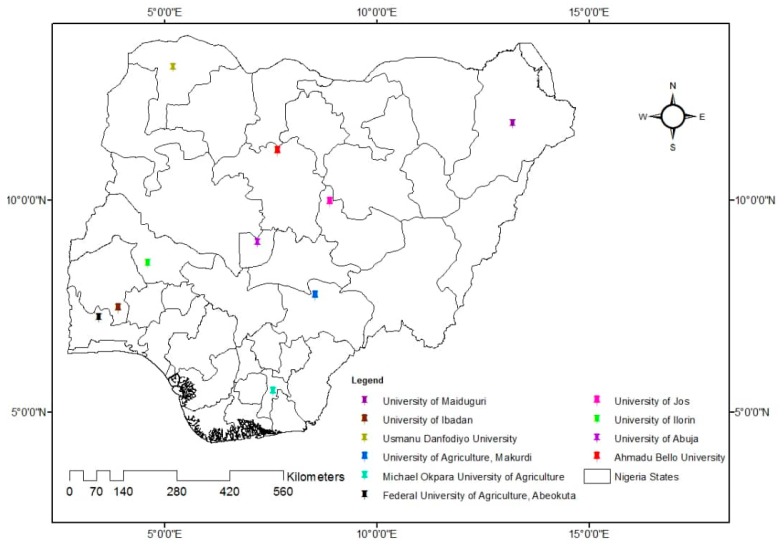
Map of Nigeria showing university locations of the participating students.

Undergraduate students in 10 of the 12 Nigerian universities that award the DVM degree were the target population. The selected universities include: University of Ilorin (UNILORIN); Federal University of Agriculture, Abeokuta (FUNAAB); University of Maiduguri, Maiduguri (UNIMAID); Usmanu Danfodiyo University, Sokoto (UDUS); University of Abuja, Abuja (UNIABUJA); Ahmadu Bello University, Zaria (ABU); University of Ibadan, Ibadan (UI); University of Agriculture, Makurdi (UAM); University of Jos, Jos (UNIJOS); and Michael Okpara University of Agriculture, Umudike (MOUAU) (Figure 1). Nigerian veterinary schools run different academic calendars based on each institution’s timelines. Due to this fact, we were unable to reach the other two veterinary schools. The universities are distributed across the six geo-political zones of Nigeria. At least, one veterinary school was sampled from each of the zones. The target population included all male and female veterinary students from year two to year six (preclinical (year two and three), para-clinical (year four), and clinical (year five and six)). No year one students were enrolled in the study. This is because year one students are in their preliminary year and do not take any core veterinary courses at that level.

### 2.2. Study Design, Sample Size, and Sampling Protocol

A cross-sectional multi-institutional questionnaire survey among veterinary students in Nigeria was carried out online from August to September 2018. The sample size formula for the cross-sectional study (random sample) [27] was used. The assumptions used in calculating the sample size were: the percentage of respondents with a poor level of knowledge was set at 50%, and the absolute precision was set at a 95% degree of confidence, with an acceptable level of error of 5%. A sample size of 384 was computed using OpenEpi [28]. To make up for non-response, 10% non-contingency was added. Therefore, a minimum of 422 veterinary students were targeted for data collection. Purposive sampling was carried out in such a way that at least 50 students were recruited at each level of veterinary education (years 2–6) across the participating universities. This means that at least 50 students were sampled from year two to year six to avoid sampling bias.

### 2.3. Questionnaire Design, Pre-Test, and Administration

This instrument comprised questions on demographics, antibiotic usage, and knowledge, attitude, and awareness on antimicrobial resistance in humans and animals. We conducted a literature search [21,29,30] and developed a 72-item survey. The questionnaire comprised seven parts: questions on demographic information, personal antibiotic use, knowledge about antibiotic use and antimicrobial resistance in humans and animals, respondents’ attitude, and awareness and contributory factors to antimicrobial resistance. Respondents were asked about their personal use of antibiotics; period of use, prescription, and advice from the doctor, and the source(s) of antibiotics used. It was believed that by asking the students about their personal antibiotic use, this could be a reflection of their overall attitude to issues of AMR. To assess respondents’ knowledge about antibiotic use and antimicrobial resistance in humans, we used 15 questions on antibiotic usage and a variety of human diseases. Participants’ knowledge of personal antibiotic usage and antimicrobial resistance in animals was assessed using 13 questions that focused on the ability of bacterial organisms to become resistant to antibiotics, the right professional to treat animals, use of antibiotics as growth promoters, antibiotic residues in animals, and the use of antibiotics to treat bacteria (pathogenic and non-pathogenic), viruses, fungi, prions, and toxins. On participants’ knowledge of the contributory factors to AMR, questions focused on the proliferation of various antimicrobial agents in circulation, use of antimicrobials in livestock and food production as growth promoters and in disease prevention in healthy animals, poor disease prevention and control strategies, under-dosage of antibiotic prescription, too-long durations of antimicrobial therapy, poor hygiene and sanitation, non-availability of new antibiotics, environmental factors and climate change, and inadequacy of drug regulatory agencies in Nigeria. These contributory factors were assessed using 10 sub-questions under one broad question with 10 check-listed answers in the online survey instrument. This question gave the opportunity for single or multiple responses simultaneously.

To assess participants’ attitudes towards AMR, we utilized a 16-item questionnaire section. Respondents’ attitudes on the effect of AMR on human and animal health and production were surveyed. Attitudinal questions about respondents having sufficient knowledge on antibiotic use for future clinical practice, need for more education on AMR at the clinical levels, poor infection control practices by veterinarians and other health care professionals as causes of the introduction and spread of AMR, following existing guidelines on the future use of antimicrobials, prescription of antibiotics in closely controlled environment, dispensing of antibiotics without prescription or over the counter, and the link between human, animal, and environmental health, in terms of AMR were asked.

Respondents were also asked about their general awareness of key terms, such as superbugs, AMR, antimicrobial stewardship, World Antibiotic Awareness Week, The Global Antimicrobial Resistance Surveillance System (GLASS), Global Antibiotic Research and Development Partnership (GARDP), Global Action Plan on Antimicrobial Resistance, and National Action Plan for Antimicrobial Resistance, Nigeria.

The questionnaire was pretested on five undergraduate DVM students from UNILORIN. Feedbacks received were used to standardize the questions before opening the online survey nationwide, for use by respondents (https://forms.gle/X9yEDCsHwq87gunS9). This online survey was accessible from 17 August 2018, 3:10:59 PM GMT+1 to 8 September 2018, 12:24:30 PM GMT+1. Informed consent was obtained. Respondents were allowed to participate in the survey voluntarily (not under group setting) and had the opportunity to withdraw from participation at any period without bias based on recommendations of the 2013 World Medical Association Declaration of Helsinki Ethical principles for medical research involving human subjects [31]. The Ethical Review Committee, Faculty of Veterinary Medicine, University of Ilorin, Ilorin approved the study (FVER/002/2019), with concurrence from other schools.

### 2.4. Data Management and Analysis

Collected data were initially summarized in Microsoft Excel 2016 and analyzed using Open Source Epidemiologic Statistics for Public Health (OpenEpi), version 3.03a [28]. Descriptive statistics were calculated for all variables and expressed as frequencies and proportions. Associations between variables were determined by the Chi-square test for categorical data or Fisher’s exact test (for 2 × 2 tables), and multivariable logistic regression models were applied where appropriate. Chi-square analysis was used to test the association between demographic factors and the outcome variables at the 95% confidence interval. Significant variables (at *p* < 0.05) were further subjected to stepwise backward likelihood multivariate logistic regression analysis to determine possible factors influencing knowledge levels among undergraduate veterinary students in Nigerian universities. Universities with <15 respondents were excluded from the multivariate logistic regression. A dependent variable was created from the thematic questions below, which were used to determine the knowledge levels of Nigerian undergraduate DVM students on antibiotic use and AMR; related questions (variables) were categorized together into various outcomes: (1) Antimicrobial resistance in humans; (2) antimicrobial resistance in animals; (3) contributory factors to antimicrobial resistance; and (4) overall knowledge on antimicrobial resistance. A numeric scoring system was used [32] to assess these outcome variables: To compute these outcome variables, respondents’ responses to questions asked were scored as “correct” or “incorrect” and transformed as “1” or “0”, respectively. The correct responses to questions in each category were added to give the general knowledge score for each of the outcome variables. Based on the scores obtained by respondents in each category; cut-off points for satisfactory scores were set and defined as scores greater than mean +1 standard deviation of the scores (Table 1). Respondents scoring above the cut-offs in each thematic area assessed were regarded as satisfactory while those with scores below were considered to have an unsatisfactory knowledge level. Specifically, for the contributory factors to AMR, each answer was treated as a response for an independent question. For each answer from a respondent, checking an option was treated as a “Yes = 1” while leaving the box unchecked was regarded as a “No = 0”. Participants’ responses on attitudinal questions were graded on a 3-point Likert scale, an agreement scale ranging from ‘1’ for “agree” to ‘3’ for “disagree”. These responses were expressed as simple percentages.

## 3. Results

### 3.1. Demographic Information

A total of 426 participants (age: 23.08 ± 2.82 years) from 10 universities across Nigeria completed the questionnaire. Table 2 displays the demographic characteristics of all respondents. The majority of respondents were male (62%) and single (93.7%). A good proportion of the respondents are in clinical levels (year 5 and 6; 47.4%).

### 3.2. Personal Antibiotic Usage Pattern among Veterinary Students

The pattern of antibiotic usage among veterinary undergraduate students in Nigeria is presented in Table 3. Generally, the veterinary students reported good personal use of antibiotics. “Personal antibiotic usage” in this context refers to antibiotics used by the veterinary students for themselves during illnesses, infection, or health, and not to treat animals; it should be understood that these students are not yet permitted to treat animals, as per professional regulations. Over half of the respondents (64.6%) reported having used antibiotics in the last six months. Respondents reported adhering to responsible personal antibiotic use by getting antibiotics from a doctor’s prescription and advice (60.7%), and these individuals sourced the antibiotics from a medical store/pharmacy (88.1%). However, few respondents believed that it is okay to use antibiotics that were given to a friend or family member, as long as they were used to treat the same illnesses or in the alternative, purchase and use previous used/recommended antibiotics for conditions presenting similar symptoms (Table 3).

### 3.3. Knowledge and Factors Associated with AMR among Respondents

On the knowledge scores of students on AMR, general knowledge scores of AMR were low and ranged from 11 to 37 (Table 1). Overall, these students (60.0%) largely demonstrated unsatisfactory knowledge scores of AMR. They also scored below average (33.2%) on knowledge of contributory factors to AMR. Multivariate logistic regression analysis revealed factors associated with knowledge of AMR among Nigerian veterinary students (Table 4 and Table 5). Students (50.0%) between 22 and 26 years were four times more likely to have satisfactory overall knowledge on AMR (OR: 3.57; 95% confidence interval (CI): 2.21, 5.77; *p* < 0.001) than other age categories (Table 4). In the institution category, the number of students from three universities (UDUS, FUUNAB, and UI) outperformed other institutions in the overall satisfactory knowledge scores. Undergraduate veterinary students from UNIMAID (OR: 0.45; 95% CI: 0.22, 0.93; *p* = 0. 043) and UNIABUJA (OR: 0.09; 95% CI: 0.02, 0.39; *p* < 0.001) were two times and 11 times less likely to possess satisfactory knowledge of AMR than students in other universities in Nigeria. Satisfactory levels of knowledge of AMR tend to increase relative to the year of study of respondents (two < three < four < five < six) (Table 5). Clinical level students (particularly those in year 6) were more likely to have satisfactory knowledge on antibiotic use and antimicrobials in humans (OR: 9.32; 95% CI: 3.45, 25.15; *p* < 001) and animals (OR: 14.16; 95% CI: 1.86, 107.50; *p* < 0.001). These students were also more likely to be aware of the contributory factors of AMR prevalence (OR: 3.25; 95% CI: 1.48, 7.12; *p* = 0.003), and have general knowledge of AMR (OR: 13.21; 95% CI: 4.88, 35.75; *p* < 0.001) than students in other levels.

### 3.4. The Attitude of Respondents to AMR

**Figure 2:**
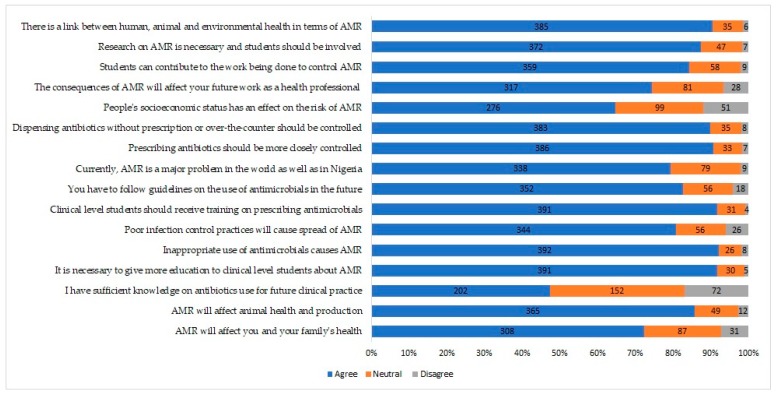
The attitude of Nigerian veterinary students to antimicrobial resistance (*n* = 426).

Respondents in this survey demonstrated positive attitudes toward AMR control (Figure 2). However, less than half (47.4%) of the participants agreed that they have sufficient knowledge of prescription for future clinical practice. The majority (>87%) agreed: that it is necessary to give more education to clinical-level students on AMR and prescribing antimicrobials before graduation; dispensing antimicrobials without prescription or over-the-counter should be closely monitored, discouraged and controlled; conducting research on AMR must essentially be with the involvement of the students; and promoting a link within human, animal, and environmental health in terms of AMR.

### 3.5. AMR Awareness among Veterinary Students

With regard to the key terms and popular statements used in AMR fora, the awareness level among veterinary students appeared low (Table 6). Only 180 (42.3%) of the respondents were aware of the term “AMR”, and less than a quarter were aware of “superbugs”, “antimicrobial stewardship”, “Global Antimicrobial Resistance Surveillance System”, “Global Antibiotic Research and Development Partnership”, and the “Global Action Plan on Antimicrobial Resistance”. More than three-quarters of respondents were unaware of the “National Action Plan for Antimicrobial Resistance, Nigeria”.

## 4. Discussion

This multi-institutional national survey investigated the knowledge and attitude of undergraduate veterinary students on antibiotic use and antimicrobial resistance with key findings. It provides an insight into the importance of possible points of intervention. Whereas veterinary students demonstrated good personal use of antibiotics, the majority had an unsatisfactory level of knowledge of AMR. Veterinary students’ academic level of study and age groupings are important factors associated with a satisfactory knowledge of AMR. It should be understood that the higher-level classes roughly correlated with higher ages and specific training in pharmacology, microbiology, and medicine, and these may have influenced the observations above. Although the respondents demonstrated positive attitudes toward tackling AMR, their awareness of key terms and concepts of AMR is low. These findings are key to understanding the situation of antibiotic use in humans and animals as well as important to evaluate knowledge of AMR among veterinary students in Nigeria. Since these students are the future prescribers for the nation’s veterinary (production and health) and food industries, they remain important stakeholders who will be playing pertinent roles in combating AMR in food animals and the environment in the future.

While over half of the respondents (64.6%) reported that the last time they used antibiotics was over six months back, based on these self-reports, the awareness of compliance with proper antibiotic usage in humans is heightened. Previously, undergraduate pharmacy students in Sri Lanka have similarly demonstrated a high degree of compliance with antibiotic use [30]. In the UK, over a third of human and animal health students were reported to have used oral antibiotics in the previous year [21]. Whether this heightened compliance is associated with training received in the field of medicine, veterinary medicine, and pharmacy has not been explored, but anecdotal evidence suggests that such knowledge will influence disposition to antibiotic usage. Besides, non-medical students in Nigeria were found to have low knowledge of antibiotic use and resistance based on previous studies [33,34]. Similar to our finding, some students in the UK were reported to obtain antibiotics from friends or family or started treatment from leftover antibiotics from a previous supply [21].

The gender distribution of respondents to the questionnaire in this survey corresponds to the gender aggregation of students of veterinary medicine in Nigerian universities. Overall, the surveyed students demonstrated an unsatisfactory level of knowledge of AMR, particularly in areas of knowledge of the use of antibiotics, AMR in humans and animals, and on the contributory factors to incidences of AMR. These findings signify a deficiency in aspects of the clinical training curricula, especially microbiology, pharmacology, and medicine, or lack of integration of knowledge as well as poor exposure of Nigerian veterinary students to associated outcomes of AMR. Comparably, in another survey, low knowledge about AMR was documented among final year veterinary students [26]

It was not surprising that the senior-level students possess higher knowledge levels on AMR compared to others. In this study, satisfactory knowledge of AMR increased with an increasing academic year of the veterinary studentship. Clinical-level students (particularly those in year six) were generally better than the other students. However, more year 5 students (34.5%) had satisfactory knowledge scores of AMR in animals than year 6 students (22.4%). A possible reason could be that year 5 students have a better memory of pharmacology since in most veterinary schools in Nigeria, pharmacology lessons are concluded in year 5 with clinical or applied pharmacology. Previous evaluation, even among the senior-level non-medical students, showed that they have appreciable knowledge on AMR than those in lower levels [33]. Sakeena et al. [30] reported that senior pharmacy students demonstrated a significantly better understanding of antibiotics and AMR when compared to junior pharmacy students in Sri Lanka. This is also similar to the findings of Hardefeldt et al. [25] in a national survey of Australian veterinary students. It can be inferred that greater exposure to the prudent use of antimicrobials during clinical teaching in the final year of veterinary school may lead to greater awareness and perceptions of AMR [25]. Efforts should also be intensified to expose veterinary students in Nigeria to AMR concepts in the preliminary years of their training. Undergraduate education is likely to be the main source of improving knowledge of antibiotics, AMR, and related terminology among health professionals as previously demonstrated in Sri Lanka [30]. Students between the ages of 22 and 26 years were four times more likely to have satisfactory knowledge on AMR in this survey, and this should translate to influence on rational use and application of antimicrobials. Students in this age category are most likely to have done preliminary years of study and maybe in the para-clinical (year four) or clinical (year five and six) years of study wherein they are exposed to pharmacology, microbiology, and related fields that may influence AMR awareness. Younger students are more informed about AMR. Expectedly, veterinary students, who are future prescribers will play an important role in the control of AMR. The observation that only a few students from three universities had satisfactory knowledge of AMR calls for serious concern. We do not have adequate information as regards the differences in curricula used to teach veterinary students across Nigerian veterinary schools, but it is expected that curricular harmonization and standardization should exist among veterinary schools in Nigeria. What is certain is that the Veterinary Council of Nigeria—the body that regulates veterinary training and practice in the country—sets a benchmark for curriculum development and use in veterinary training. We also could not confirm if AMR was adequately emphasized for teaching in the benchmark. However, our study suggests that the benchmark should be honed up, monitored, and periodically evaluated, to emphasize the teaching of AMR across years of study in veterinary schools in Nigeria. AMR education in all Nigerian veterinary schools should be reworked, reoriented, and focused to meet these identified gaps.

Respondents to our survey exhibited positive attitudes toward AMR control. A similar observation has been observed among undergraduate paramedical students [29]. The respondents agreed that it is necessary to give more education to clinical-level students on AMR, and on the prescription of antimicrobials before graduation; dispensing of antimicrobials and control of over-the-counter access to medication; and on the conduct of research on AMR with the involvement of students. However, less than half (47.4%) of the participants agreed that they have sufficient knowledge on antibiotic use for future clinical practice. Some Australian veterinary students similarly opined that more education is needed in veterinary pharmacology [25]. Surveyed medical students in the United Kingdom, France, and the United States of America have also demonstrated the need for more education on AMR [29,35,36,37,38,39]. Preliminary-level introduction and exposure to training on AMR in Nigerian veterinary schools at lower levels of training will improve students’ appreciation and understanding of antibiotic use and AMR. Also, having a one health perspective on AMR is important since it is a trans-national and global issue linking humans, animals, and the environment.

On key terms in AMR, only 42.3% of respondents were aware of the term “AMR”. This is confirmed by the previous observation of Anyanwu et al. [26]. Similar low scores (approximately 25%) were observed for “superbugs”, “antimicrobial stewardship”, “Global Antimicrobial Resistance Surveillance System”, “Global Antibiotic Research and Development Partnership”, and the “Global Action Plan on Antimicrobial Resistance”. For the “National Action Plan for Antimicrobial Resistance, Nigeria”, three-fourths of respondents were unaware. This low level of awareness on AMR could be attributable to the lack of in-depth AMR education in Nigerian veterinary schools [26] and deficiencies in veterinary curricula [38,39]. Furthermore, the unexpectedly low national attention accorded to AMR in food animals and the environment could contribute to the low awareness observed. Comparably, fewer than half of all students (44%) had heard of either “antimicrobial stewardship” or “antibiotic stewardship” in a survey of human and animal health students in the UK [21].

A major limitation of this study was the use of an online questionnaire tool for data collection, which may be associated with enrolment bias. However, the overall response rate is sufficient to eliminate or reduce this bias. Because of the peculiar situation of some institutions, some students could have had limited access to the online questionnaire due to poor availability and limited access to the internet, and this possibly affected the uneven distribution of respondents from various schools. Also, it is harder to draw probability samples based on e-mail addresses or website visitations as well as a lack of trained interviewers to clarify and probe responses. Nevertheless, it was believed that the pre-testing before actual data collection should improve the accuracy and internal quality control of the tool. The logistic regression model was run with year two students serving as the reference against other years of study. Only one year two student had satisfactory knowledge of AMR in animals, resulting in a small sample size of year two students with satisfactory knowledge. Thus, this affected the outcome resulting in a wide confidence interval for students in higher years of study (4–6) with more satisfactory knowledge scores of AMR in animals. Also, wording options, such as “inadequacy of drug regulation agencies”, sounds extremely bias, possibly influencing students to click on them. However, only about 58% of respondents picked these options. Thus, the bias was unpronounced as envisaged.

## 5. Conclusions

This study expounded the unsatisfactory knowledge level of AMR issues existing among veterinary students in Nigeria, yet willingness to improve was observed. Identified knowledge, attitude, and awareness of AMR gaps should be targeted by veterinary schools in combatting AMR issues in Nigeria.

## Figures and Tables

**Table 1:** Description of scores (outcomes) obtained by respondents (*n* = 413).

Outcomes	Maximum Obtainable Score	Scores Obtained by Respondents		Mean ± SD	Satisfactory *n* (%)	Unsatisfactory *n* (%)
		Lowest	Highest			
Antibiotics and antimicrobial resistance in humans	15	5.00	15.00	11.33 ± 2.25 ^1^	146 (35.4)	267 (64.6)
Antibiotics and antimicrobial resistance in Animals	13	4.00	13.00	9.97 ± 1.69 ^1^	72 (17.4)	341 (82.6)
Contributory factors to antimicrobial resistance	10	0.00	10.00	5.24 ± 2.69 ^1^	137 (33.2)	276 (66.8)
Overall knowledge of antimicrobial resistance	38	11.00	37.00	26.55 ± 4.93 ^1^	166 (40.0)	248 (60.0)

^1^ Satisfactory scores = scores > mean + 1 standard deviation.

**Table 2:** Demographic distribution of Nigerian veterinary students participating in the survey (*n* = 426).

Demographic Factors	*n* (%)
**Gender**	
Male	264 (62.0)
Female	162 (38.0)
**Age (year)**	
17–21	141 (33.1)
22–26	237 (55.6)
27–31	48 (11.3)
**Level of study**	
Year 2	56 (13.1)
Year 3	84 (19.7)
Year 4	84 (19.7)
Year 5	86 (20.2)
Year 6	116 (27.2)
**University**	
UNILORIN	79 (18.5)
FUNAAB	16 (3.8)
UNIMAID	64 (15.0)
UDUS	40 (9.4)
UNIABUJA	31 (7.3)
ABU	28 (6.6)
UI	26 (6.1)
UAM ^1^	5 (1.2)
UNIJOS ^1^	8 (1.9)
MOUAU	129 (30.3)

UNILORIN—University of Ilorin, Ilorin; FUNAAB—Federal University of Agriculture, Abeokuta; UNIMAID—University of Maiduguri, Maiduguri; UDUS—Usmanu Danfodiyo University, Sokoto; UNIABUJA—University of Abuja, Abuja; ABU—Ahmadu Bello University, Zaria; UI—University of Ibadan, Ibadan; UAM—University of Agriculture, Makurdi; UNIJOS—University of Jos, Jos; MOUAU—Michael Okpara University of Agriculture, Umudike. ^1^ Excluded from univariate and multivariate analysis because *n* <15.

**Table 3:** Personal use of antibiotics of Nigerian veterinary students (*n* = 426).

Parameters	*n* (%)
Last antibiotic use	
In the last month	167 (39.2)
In the last 6 months	108 (25.4)
In the last year	47 (11.0)
More than a year ago	29 (6.8)
Never	15 (3.5)
Can’t remember	60 (14.1)
Getting antibiotics from a doctor’s prescription	
Yes	250 (60.7)
No	143 (34.7)
Can’t remember	18 (4.4)
On that occasion, did you get advice from a doctor, nurse or pharmacist on how to take them?	
Yes	290 (70.4)
No	104 (25.2)
Can’t remember	18 (4.4)
On that occasion, where did you get the antibiotics?	
Medical store/Pharmacy	363 (88.1)
Stall/hawker	7 (1.7)
Friends/Family member	18 (4.4)
I have them saved up from previous time	10 (2.4)
Somewhere/someone else	14 (3.4)
When do you think you should stop taking antibiotics once you have begun treatment?	
When you feel better	44 (10.7)
When you have taken all antibiotics as directed	359 (87.1)
Don’t know	9 (2.2)
It is okay to use antibiotics that were given to a friend or family member, as long as they were used to treat the same illnesses	
False	297 (72.1)
True	89 (21.6)
Don’t know	26 (6.3)
It is okay to buy the same antibiotics, or request these from a doctor if you are sick and they helped you get better when you had the same symptoms before?	
False	220 (53.4)
True	158 (38.3)
Don’t know	34 (8.3)

**Table 4:** Likelihood multivariate logistic regression analysis of the age of veterinary students as a factor associated with antimicrobial resistance in Nigeria.

Age (Years)	Antibiotics and Antimicrobial Resistance in Humans					
	Unsatisfactory (%)	Satisfactory (%)	*p* Value (χ2)	OR	95%CI	*p* Value
17–21	107 (85.0)	30 (15.0)	0.000	1.00	-	-
22–26	135 (58.7)	95 (41.3)		2.51	1.55, 4.07	<0.001 *
27–31	25 (54.3)	21 (45.7)		2.99	1.48, 6.08	0.004 *
	Antibiotics and antimicrobial resistance in animals					
	Unsatisfactory (%)	Satisfactory (%)				
17–21	122 (89.1)	15 (10.9)	0.049	1.00	-	-
22–26	183 (79.6)	47 (20.4)		2.09	1.12, 3.90	0.025 *
27–31	36 (78.3)	10 (21.7)		2.26	0.94, 5.46	0.118
	Contributory factors to antimicrobial resistance					
	Unsatisfactory (%)	Satisfactory (%)				
17–21	105 (76.6)	32 (23.4)	0.004	1.00	-	-
22–26	138 (60.0)	92 (40.0)		2.19	1.36, 3.52	0.001 *
27–31	33 (71.7)	13 (28.3)		1.29	0.61, 2.73	0.629
	Overall knowledge of antimicrobial resistance					
	Unsatisfactory (%)	Satisfactory (%)				
17–21	107 (78.1)	30 (21.9)	0.000	1.00	-	-
22–26	115 (50.0)	115 (50.0)		3.57	2.21, 5.77	<0.001 *
27–31	26 (56.5)	20 (43.5)		2.74	1.35, 5.58	0.009 *

*—significant at *p* < 0.05; χ_2_—Chi-square; CI—confidence interval; OR—odds ratio.

**Table 5:** Likelihood multivariate logistic regression analysis of the level of study of veterinary students as a factor associated with antimicrobial resistance in Nigeria (*n* = 413).

Year of Study	Antibiotics and Antimicrobial Resistance in Humans					
	Unsatisfactory (%)	Satisfactory (%)	*p* Value (χ2)	OR	95% CI	*p* Value
Two	45 (90.0)	5 (10.0)	0.000	1.00	-	-
Three	64 (81.0)	15 (18.9)		2.11	0.71, 6.22	0.259
Four	57 (67.9)	27 (32.1)		4.26	1.52, 11.95	0.005 *
Five	44 (52.4)	40 (47.6)		8.12	2.96, 22.65	<0.001 *
Six	57 (49.1)	59 (50.9)		9.32	3.45, 25.15	<0.001 *
	Antibiotics and antimicrobial resistance in animals					
	Unsatisfactory (%)	Satisfactory (%)				
Two	49 (98.0)	1 (2.0)	0.000	1.00	-	-
Three	75 (94.9)	4 (5.1)		2.61	0.28, 24.08	0.709
Four	71 (84.5)	13 (15.5)		8.97	1.14, 388.90	0.020 *
Five	55 (65.5)	29 (34.5)		25.89	3.39, 196.80	<0.001 *
Six	90 (77.6)	26 (22.4)		14.16	1.86, 107.50	<0.001 *
	Contributory factors to antimicrobial resistance					
	Unsatisfactory (%)	Satisfactory (%)				
Two	40 (80.0)	10 (20.0)	0.000	1.00	-	-
Three	64 (81.0)	15 (19.0)		1.00	0.41, 2.42	>0.999
Four	60 (71.4)	24 (28.6)		1.60	0.69, 4.16	0.371
Five	48 (57.1)	36 (42.9)		3.00	1.33, 6.79	0.011 *
Six	64 (55.2)	52 (44.8)		3.25	1.48, 7.12	0.003 *
	Overall knowledge of antimicrobial resistance					
	Unsatisfactory (%)	Satisfactory (%)				
Two	45 (90.0)	5 (10.0)	0.000	1.00	-	-
Three	65 (82.3)	14 (17.7)		1.94	0.65, 5.76	0.343
Four	56 (66.67)	28 (33.33)		4.50	1.61, 12.60	0.003 *
Five	35 (45.2)	49 (54.8)		12.60	4.50, 34.96	<0.001 *
Six	47 (40.52)	69 (59.48)		13.21	4.88, 35.75	<0.001 *

*—significant at *p* < 0.05; χ2—Chi-square; CI—confidence interval; OR—odds ratio.

**Table 6:** Awareness level of veterinary students on terms relating to antimicrobial resistance (*n* = 426).

Terms	No (%)	Yes (%)
AMR	246 (57.7)	180 (42.3)
Superbugs	353 (82.9)	73 (17.1)
Antimicrobial stewardship	370 (86.9)	56 (13.1)
World Antibiotic Awareness Week	272 (63.8)	154 (36.2)
Global Antimicrobial Resistance Surveillance System (GLASS)	324 (76.1)	102 (23.9)
Global Antibiotic Research and Development Partnership (GARDP)	343 (80.5)	83 (19.5)
Global Action Plan on Antimicrobial Resistance	349 (81.9)	77 (18.1)
National Action Plan for Antimicrobial Resistance, Nigeria	309 (72.5)	117 (27.5)
